# Metastasis Update: Human Prostate Carcinoma Invasion via Tubulogenesis

**DOI:** 10.1155/2011/249290

**Published:** 2011-06-21

**Authors:** Raymond B. Nagle, Anne E. Cress

**Affiliations:** ^1^Department of Pathology, The Arizona Cancer Center, The University of Arizona, Tucson, AZ 85724, USA; ^2^Department of Cellular and Molecular Medicine, The University of Arizona, Tucson, AZ 85724, USA

## Abstract

This paper proposes that human prostate carcinoma primarily invades as a cohesive cell collective through a mechanism similar to embryonic tubulogenesis, instead of the popular epithelial-mesenchymal transformation (EMT) model. Evidence supporting a tubulogenesis model is presented, along with suggestions for additional research. Additionally, observations documenting cell adhesion molecule changes in tissue and stromal components are reviewed, allowing for comparisons between the current branching morphogenesis models and the tubulogenesis model. Finally, the implications of this model on prevailing views of therapeutic and diagnostic strategies for aggressive prostatic disease are considered.

## 1. Introduction

Most pathologists recognize prostate cancer as a series of polarized glandular structures lacking basal cells and varying in differentiation from lumen forming tubules to solid cords. These morphological observations are consistent with an invasion model in which cohesive groups of cells bud off from an in situ precursor lesion such as high-grade prostate intraepithelial neoplasia (HGPIN). Recent molecular marker expression studies are also consistent with this view. However, a prevalent view of prostate cancer invasion depicts single tumor cells invading the surrounding stroma, preceding vascular intravasation and dissemination. This widely held view of metastasis of epithelial cancers involves an epithelial-mesenchymal transformation (EMT) [1]. EMT of the malignant cells at the primary tumor allows for a motile invasive single-cell phenotype [1–4]. 

EMT is associated with the loss of epithelial-specific E-cadherin from the adheren junctions and a switch from the expression of keratins as the major intermediate filament to the mesenchymal intermediate filament, vimentin [5]. While this concept may be formally possible in epithelial cancers, it is rarely observed in prostate cancers except in the relatively rare Gleason Grade 5 tumors. In fact, others have noted that EMT in cancer invasion is not universally observed [6–8]. Additionally, some models state that, in the absence of EMT inducing signals, tumor cells may also reverse the process and undergo a mesenchymal to epithelial transition (MET) [9, 10]. This transient nature is proposed to explain why metastatic cells morphologically resemble primary tumor cells. An alternative possibility is that the cancer phenotype does not change and, therefore, requires no companion MET process. We propose that human prostate cancer invasion is an EMT-independent event. The invasive collective of tumor cells remain epithelial in nature—and, therefore, do not require a shift back to the epithelial phenotype. This review will challenge the applicability of the EMT concept for prostate cancer and offer an alternative idea: primary prostate cancers invade by a process similar to embryonic tubulogenesis. 

## 2. Prostate Cancer Morphology

**Figure 1:**
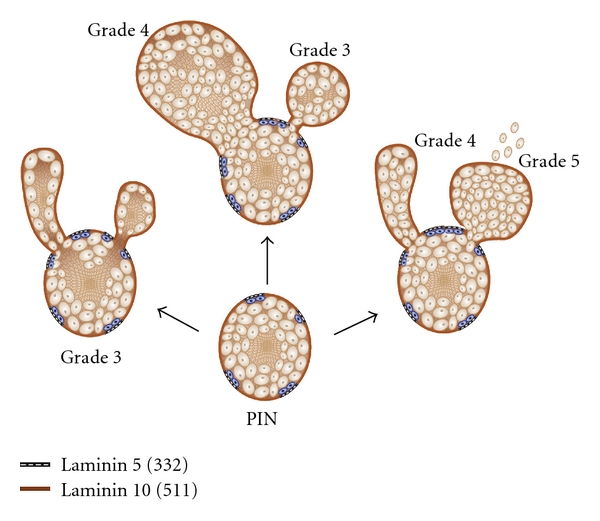
Tubulogenesis model of prostate cancer invasion. High-grade prostatic intraepithelial neoplasia (PIN) gives rise to various degrees of polarity and differentiation of cellular buds. PIN lesions are glandular-type structures characterized by gaps of laminin 10 (brown bar, laminin 10 (511)) and sporadic retention of basal cells (blue) attached to a laminin 5 matrix (laminin 5, 332). Three different patterns of spread (arrows) arise from PIN lesions. Note complete polarity and lumen formation (grade 3), partial lumen formation in cribiform lesion (grades 3-4 depending on size) and lack of lumen formation (grade 5). Importantly, budding occurs in areas where basal cells are lost, and the basal lamina lacks laminin 5 (332); the invasive budding clusters of cells are exposed to laminin 10 (511).

A modified grading system based upon Gleason scoring is used to describe prostate cancer morphology [11]. The majority of low Gleason Grade lesions and even Gleason Grade 4 lesions arise from high-grade prostatic intraepithelial neoplasia (PIN) lesions and appear as invasive tubular structures (Figure 1). 

Invasive tubular structures persist in lymph node metastatic lesions, as judged by prominent E-cadherin expression [12, 13], suggesting that prostate carcinoma invades by collective cell migration (see Friedl and Gilmour [14]), a process analogous to normal tubulogenesis. In embryologic tubulogenesis, coherent cells influenced by stromal factors initially migrate into the surrounding stroma as solid cords of cells. Later, lumina are formed and the cells develop polarity with their luminal surfaces facing a lumen and with their basal surfaces resting on a basal lamina [15]. In simple Grade 3 lesions, the polarity is complete. In cribiform Grade 3 and 4 lesions, the polarity is deranged with multiple lumina. In Grade 4, there is solid cord-like lesions form that lacks any lumina. The normal morphological alterations and modifications of the prostate gland yield important clues to the molecular events involved in the deregulation of the gland during cancer progression. In particular, prostate cancer tubulogenesis occurs in areas where the basal cells are lost and the basal lamina lacks laminin 332 (Figure 1). 

### 2.1. The Relationship of Prostate Glands to the Surrounding Stroma

The prostate gland, under the influence of androgen, develops from the endoderm-derived urogenital sinus to form branched tubuloalveolar glands [16]. These normal prostate glands are composed of two cell types, the basal cell and the secretory luminal cells [16]. The normal glands are surrounded by a delicate basal lamina containing laminins 111/121, 211, 332, and 511/521, as well as collagen IV and collagen VII [17]. The basal cells attach to this substratum through a number of integrins: *α*2, *α*3, *α*4, *α*5, *α*6, coupled with *β*1 and *α*v*β*3 [18]. A dominant attachment occurs through hemidesmosomes via the *α*6*β*4 integrin, an essential gene product, interacting at the c-terminal ends with anchoring filaments (laminin 332) that, in turn, interact with anchoring fibers (Collagen VII) [19]. Loss of the *α*6*β*4 integrin function in normal epithelial tissues results in blistering diseases, indicating its essential role [20]. The architecture and assembly of ECM molecules in embryonic spaces provides a morphogenetic language or code that can promote or restrict cell movements and determine cell fate [21, 22]. In human prostate cancer, loss of *α*6*β*4 integrin and type VII collagen is a universal feature [13, 18, 19, 23, 24]. In preclinical models, normal prostate cells have a robust DNA damage response dependent upon laminin [25]; early loss of the laminin receptor, *α*6*β*4 expression, promotes tumor progression [26]. In the model proposed here, the documented loss of a dominant adhesion structure is permissive for the cohesive budding of cell clusters into the stroma.

 The luminal cells are thought to arise from stem-type cells within the basal cell population [27]. The luminal cells are primarily secretory, express androgen receptors, and produce the proteins of the seminal fluid, including prostate specific androgen (PSA). Mitotic errors during intermediate stages of luminal cell development have been postulated as a possible origin for human prostate cancer [28]. In addition, recent work has indicated that luminal cells as compared to basal cells appear defective in their ability to invoke a DNA damage response [29].

Taken together, these observations suggest that loss of a dominant adhesion structure permits budding of cell clusters that are more susceptible to fixed DNA damage. In this context, we note that an accumulation of fixed DNA damage has been previously reported in human prostate cancer tissue [30]. Further, the loss of the normal glandular structure and the loss of fundamental positional cues would provide extracellular signals for invasive budding within a new environment, rich in laminin 511, an essential molecule in development that determines cell fate.

### 2.2. Changes during Prostate Cancer Progression

In PIN (prostatic intraepithelial neoplasia) lesions, cells with enlarged nuclei and often prominent nucleoli proliferate within the lumen, enlarging the glands and eventually causing the basal cell layer to become attenuated, resulting in continuity gaps. Interestingly, where the basal cells persist, the integrin expression and the hemidesmosomes also persist, including the underlying basal lamina that expresses laminin 332 [31]. In the gaps where the basal cells are lost, laminin 332 and the protein elements of the hemidesmosome are missing [18, 19, 23, 24]. The cells in these gap areas are attached in the gland via integrins *α*6*β*1 and *α*3*β*1 [32] and are reactive with an underlying basal lamina expressing laminin 511, a laminin important for epithelial tubulogenesis [33, 34] but not laminin 332. The cells making up the PIN lesions express a mixture of basal cell and luminal cell proteins, further suggesting origination in faulty mitosis [28] or defective DNA damage repair [29]. Analysis of a variety of morphologic nuclear features showed that these cells are very similar to invasive carcinoma cells and are already showing signs of genetic instability with a rate of aneuploidy similar to invasive carcinoma [35, 36]. 

**Figure 2:**
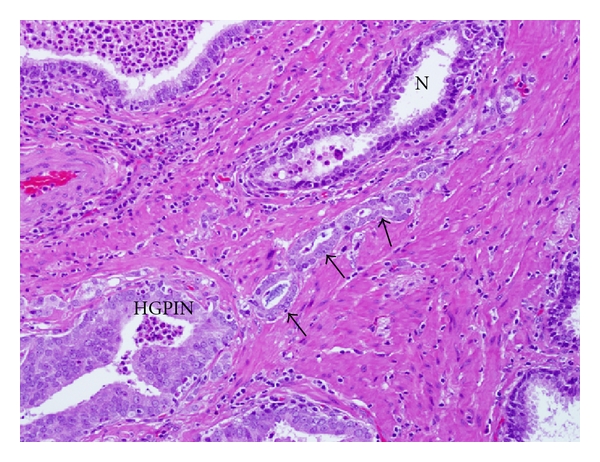
High grade PIN lesion showing budding invasive structure. PIN lesion (HGPIN) progressively changes into proximal lumen formation (arrows) and a distal solid cord of tumor cells. Normal prostate gland (N) is shown for comparison. H&E X400.

Recent studies have shown that approximately 16% of PIN lesions show the rearrangement of the ETS-related gene (ERG) [37, 38]. TMPRSS-ERG (transmembrane serine protease) gene fusions are associated with the loss of *α*6*β*4 integrin expression, the known regulator of hemidesmosome assembly. Numerous studies have associated early invasive carcinoma with these PIN lesions [27, 39–42]. Others have shown a discrepancy between the occurrence of high-grade PIN (HGPIN) and carcinoma, suggesting that HGPIN is not a precursor to invasive carcinoma [43, 44]. However, serial sectioning of HGPIN reveals invasive tubular structures arising from the PIN lesions (Figure 2, arrows). 

**Figure 3:**
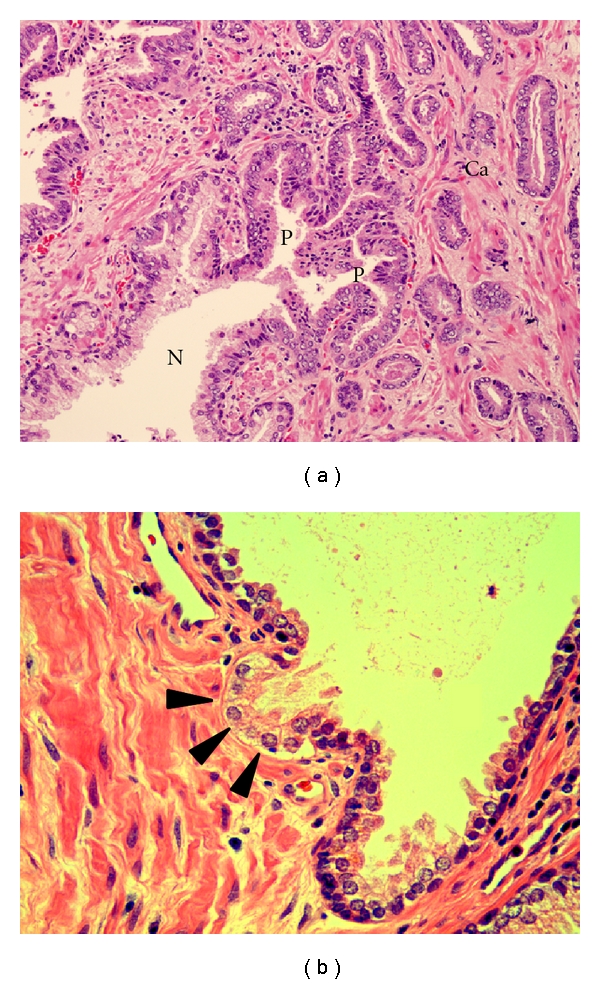
Progressive morphological features of tubulogenesis in human prostate cancer. (a) is a tissue section stained with H&E X 200 and shows the transition of a normal prostate gland (N) to high-grade PIN (P) which has budded into invasive low-grade carcinoma (Ca). (b) is a tissue section stained with H&E X 400 demonstrating a prostate gland showing an early bud (arrows) of atypical cells. Note the absence of basal cells in the budding lesion.

The invasive cell clusters arise from gap regions that lack basal cells. The early detection of an invasive cell cluster is observed as a budding of atypical cells into the stroma (Figure 3). Of particular note is the lack of basal cells within the lesion (Figure 3, arrows) which corresponds to the known loss of dominant adhesion structures. As stated earlier, the invasive cells have lost hemidesmosomes and have a restricted *α*6*β*1, *α*3*β*1 integrin expression [19]. 

**Figure 4:**
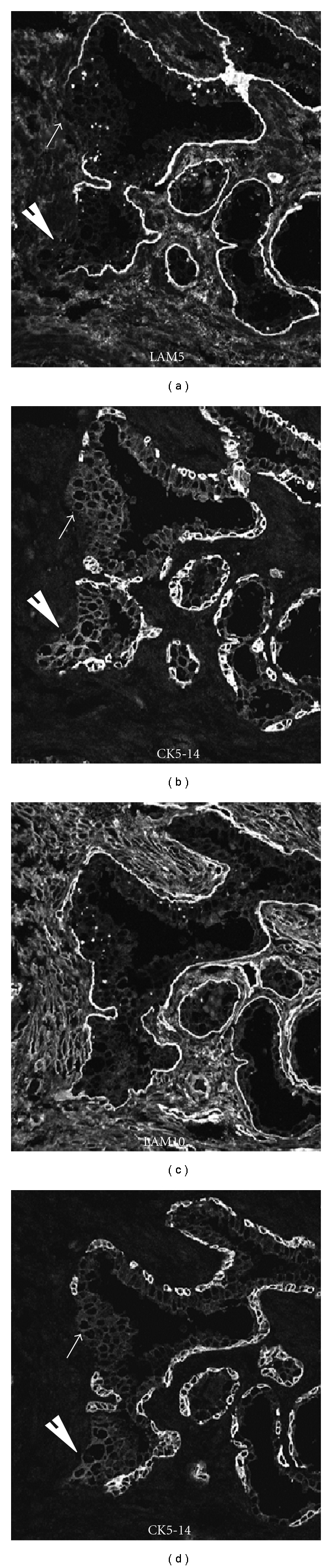
Budding lesions are devoid of basal cells and lack laminin 5 deposition and become exposed to laminin 10. Serial sections containing cell clusters (white arrows) were stained either for laminin 332 (LAM5) or laminin 511 (LAM10) and the basal cell-specific marker, cytokeratin 5 and 14 (CK5-14).

The lack of basal cells in the budding cancer clusters is confirmed by a loss of cytokeratin 5 and 14 expression (basal cell markers) and the corresponding loss laminin 332 (laminin 5) expression in the basal lamina, as observed in serial sections shown in Figure 4. Of particular interest is that while laminin 332 expression is lost in the budding lesion, another form of laminin, laminin 511 (laminin10), is abundant in the microenvironment, surrounding the glands vessels and prominently expressed in the stroma (Figure 4). Laminin 511 (LAM 10) is a potent morphogen essential for embryonic development and governs cell fate [34]. As stated earlier, invasive cancers express *α*6*β*1 and *α*3*β*1, laminin 511 binding integrins. 

Further studies utilizing in situ hybridization techniques have shown that all three of the mRNAs encoding the three laminin 332 chains are present and have normal sequences, a finding that suggests the loss of protein expression is controlled at the translational level [24, 45]. These cells are polarized and have intact tight junctions as well as intact zonula adherens [46, 47]. In less differentiated grades, they may form cribiform glandular structures or solid trabecular structures lacking lumens. 

### 2.3. Relationship of Prostate Cancer Invasion to Tubulogenesis

These early invasive events in which proliferating groups of cells maintain cellular adhesion and reestablish tubular structures closely resemble embryologic tubulogenesis. Knowledge of collective cellular migration (reviewed in Friedl and Gilmour [14]) is derived from several areas of embryology including the study of border cell migration in Drosophila oogenesis [48], tracheal branching morphogenesis in insects [49, 50], mammary gland development [51, 52], and lateral line organogenesis in zebra fish [53]. From studies in these and other systems, a concept of tubulogenesis has arisen in which a placode of cells in an originating epithelium gives rise locally to cells that migrate as a cohesive mass in response to promigratory and polarity-preserving signals produced by neighboring stromal cells. In order for these events to occur, there must be cell-cell cohesion, maintenance of polarity, cytoskeleton reorganization and force generation, extracellular matrix (ECM) remodeling, and stromal signal generation. 

 Although these processes are not as clearly understood in cancer as they are in normal embryogenesis, there is accumulating evidence that the process in cancer progression is similar. It is clear from immunohistochemical studies that low-grade prostate carcinomas maintain cell cohesion through components of the tight junction including Z01, claudins and occludin (see Martin and Jiang [47]), zonula adherens (E-cadherin, B-catenin, desmosomes) [46], as well as gap junction proteins and apical adhesion molecules such as CEACAM1 (carcinoembryonic antigen-related cell adhesion molecule 1) [54]. 

**Figure 5:**
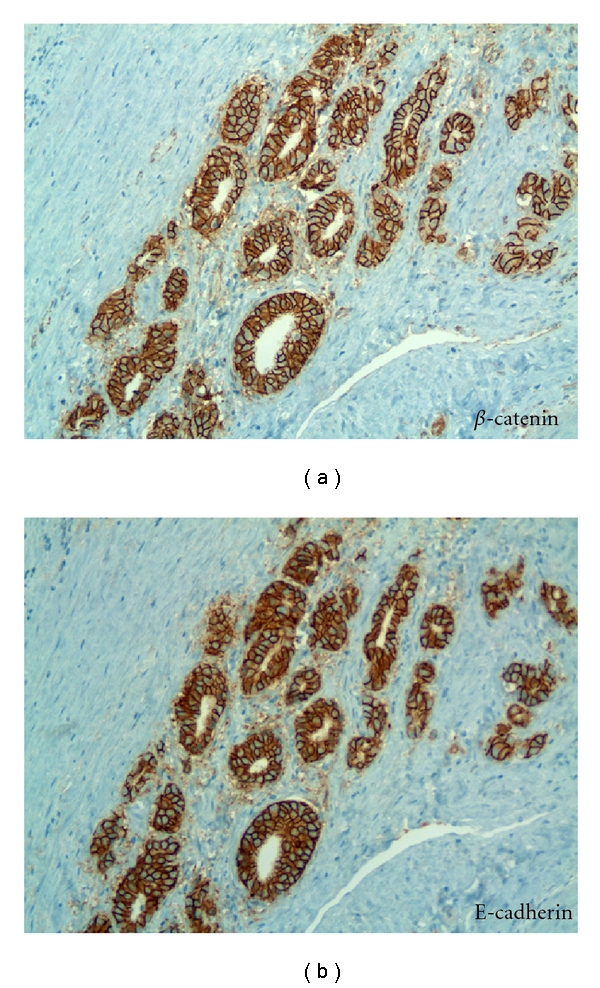
Preservation of epithelial marker expression in invasive prostate carcinoma. Serial sections of Gleason Grade 3 prostate carcinoma reacted in (a) with anti-B-catenin and (b) reacted with anti-E-cadherin. Note maintenance of intracellular adhesion and polarity in invasive carcinoma. X 200.

The maintenance of cell polarity is variable, with well-differentiated tumors forming basal-luminal polarity in the absence of basal cells. For example, E-cadherin and B-catenin are expressed in low-grade prostate adenocarcinoma (Figure 5). E-cadherin expression results in survival advantage for tumor cells [55, 56]. Specifically, E-cadherin dampens cellular motility behavior by biasing the direction of cell migration without affecting the migration rate. The results also demonstrated that there is cross-talk between E-cadherin and integrin-based adhesion complexes [57]. Integrin alpha 6 expression in human prostate carcinoma is associated with a migratory and invasive phenotype both in vitro and in vivo [58]. Taken together, these results would indicate that the preservation of E-cadherin and laminin-binding integrin expression in prostate cancer tubulogenesis could aid in the formation and direction of tubular growth. 

Several reports have shown reduced expression of E-cadherin and B-catenin with increasing Gleason grade [59–61]. Murant et al. [59] made the interesting observation that there was a reciprocal increase of B1 integrin as E-cadherin decreased. Busch et al. [54] demonstrated that occludin, a component of the tight junctions, was expressed in low-grade prostate tumor but, with polarity loss, was downregulated in Gleason Grade 4 tumors and completely lost in Grade 5 tumors. 

 Tubulogenesis results in prostate cancer cells becoming attached to a newly synthesized basal lamina. In less differentiated tumors, complex cribiform structures are formed with multiple intraglandular lumina. The invasion process in human prostate carcinogenesis is slow, and little information is available regarding changes in cytoskeleton proteins at the leading edge of the invading tubular structure, although these contractile proteins are known to be important in normal tubulogenesis [15]. 

It is also clear that there must be initial ECM degradation and regeneration of new basal lamina to support the tubular structures. Studies of invading cells in liquid culture or 3D gels demonstrate two surface metalloproteinase molecules, MTIMMP and MMP2, which degrade the ECM along the leading cells [61, 62]. Our own studies of invasion utilizing an xenograft model of DU145 human prostate cells seeded onto the murine diaphragm revealed tumor colonization of the surface. Collective cell invasion was induced when the tumor cells were permanently transfected to express the metalloproteinase MMP7 [63]. The murine diaphragm surface mimics the stroma of the prostate and contains a vascular supply, sensory and motor nerve endings, stromal fibroblasts, and muscle cells, making it a useful model environment [64]. All of these cell types are potential sources of stimulatory factors. 

**Figure 6:**
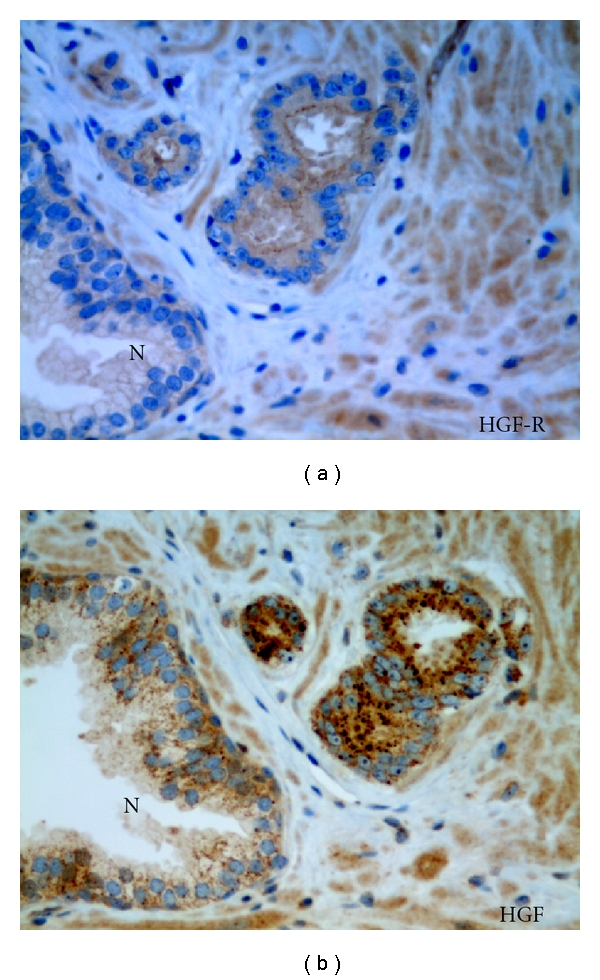
Increased expression of a morphogenic growth factor and receptor in invasive budding cancer. Serial sections of Gleason Grade 3 prostatic carcinoma and normal gland (N) reacted in (a) with anti-c-Met (aka Hepatocyte Growth Factor (HGF) receptor) and (b) reacted with anti-HGF. X 400.

Invasion of oral squamous cell carcinoma in vitro reportedly has been stimulated by paracrine SDF1 and hepatocyte growth factor produced by stromal fibroblasts driven by tumor cell-derived cytokines [65]. There is an extensive literature describing the role of hepatocyte growth factor (HGF) and its receptor c-Met in prostate cancer progression (see Hurle et al. [66]). Interaction of HGF with its receptor has been demonstrated to modulate cell proliferation, tumor cell interaction, cell migration, cell-matrix adhesion, cell invasion, and angiogenesis in prostate cancer cells (Figure 6). Other factors such as FGF and TGF-b have been also implicated in the stimulation of tumor cell invasion [3, 67]. 

Another signaling factor known to be important in normal embryonic epithelial modeling is the Wnt pathway, which is involved in cell fate specification, proliferation, polarity, and migration [68]. Both the classic pathways—involving a variety of Wnt ligands binding to the Frizzle receptor and resulting in *β*-catenin transcription—and the non-Canonical pathway [68] demonstrate activity in prostate cancer (see Yardy and Brewster [69]). Studies have shown that *β*-catenin interacts with the androgen receptor, perhaps further indicating its relevance to prostate cancer progression [70].

Cells that eventually intravasate into vessels, it seems, leave the active tips of the tubular invasive structures. Single-cell migration into vessels would represent a form of EMT, a possible late event in tubulogenesis, but this needs more detailed documentation and validation. Moreover, there is some evidence that even these intravascular cells retain cohesive properties and actually travel as small groups of attached cells [14]. A careful analysis of changes occurring at the tips of these tubular structures is likely to produce important information that may become the cornerstone of new diagnostic and therapeutic treatments aimed at preventing prostate carcinoma metastasis.

**Figure 7:**
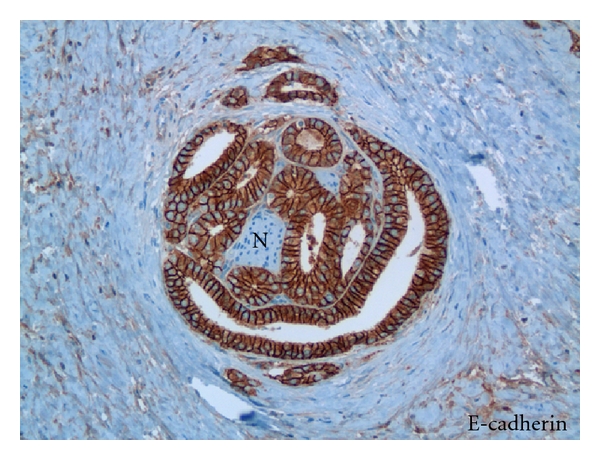
Invasive perineural prostate cancer maintains cell polarity and intracellular adherence. Tissue section of prostate carcinoma reacted with anti E-Cadherin antibody and surrounding a nerve (N). X 400.

There is considerable evidence that nerves within the peripheral zone in proximity to prostate cancer facilitate tumor penetration of the capsule [67, 71]. Perineural prostate carcinoma growth is routinely observed in areas of extra prostatic extension, where these carcinomas can maintain polarity (Figure 7) and have been observed lining up along the basement membrane. 

Invasive cancer invading stroma and then traveling along neural structures has been observed in pancreatic cancer, using serial sectioning methods to reveal tumors growing in a continuous fashion [72]. While similar studies have not been published describing this event in prostate cancer, we infer that tubular structures of invading prostate carcinoma would encounter nerve structures and then travel along these conduits finally reaching the para-prostatic connective tissue [73]. It is not clear at what juncture these cells would intravasate into vascular structures, but it is clear that perineural prostate cells are not within vessel lumens, despite growing in close proximity to lymphatic vessels. 

### 2.4. Implications of the Tubulogenesis Model of Prostate Cancer Progression

There is a pressing need for biomarkers that distinguish indolent from aggressive prostate cancer. It is estimated that 30 to 50% of men diagnosed with prostate cancer could avoid surgery or radiation (and instead be followed by active surveillance) because they have “good prognosis” tumors that are unlikely to progress [74]. Further, recent reports indicate that approximately 50% of patients that are classified as high risk do not develop metastases and 10% of patients classified as low risk develop secondary disease [75]. The critical need for biomarkers has led to integrative genomic profiling of human prostate cancer to annotate alterations corresponding to clusters of low- and high-risk disease beyond that achieved by the Gleason Score [76]. 

Taylor et al. [76], in a hallmark study, combined methods of pathologist-guided dissection with comprehensive genomic analysis and clinical outcome data. Transcriptomes were defined and copy number alterations documented in 218 prostate tumors (181 primaries and 37 metastases). Several known cancer pathways were observed in human prostate cancer, and the study revealed that nearly all metastases contained changes in P13K, RAS/RAF, and androgen receptor pathways. Independent work examining tumor cells within bone marrow revealed a loss of cell adhesion components in disseminated tumor cells as a potential harbinger of aggressive disease [75]. 

Extending the primary tumor analysis approach to understanding the signatures of invasive budding tumors, rather than analysis of the entire cancer specimen, would likely reveal aggressive subsets of tumors. Prostate cancer is multifocal, and intratumor genomic heterogeneity is a well-known phenomenon [77]. Restricting analysis to the invasive tips of the tumor may clarify the relevance of the molecular signatures for identifying aggressive disease. The inherent difficulty in distinguishing the budding cancer from the tumor epicenter will require developing improved strategies of tissue analysis. Recent studies have used a strategy of multiplexed quantum dot mapping to begin providing correlated molecular and morphological information [78]. In other studies, terminal end buds (TEB) during mammary branching morphogenesis have been microdissected, and the transcriptomes identified; specific gene signatures are associated with TEB [79]. A similar strategy could be utilized to define budding prostate cancer from the bulk of the tumor. 

In a similar fashion, the responsiveness of the tumor to therapeutic approaches, such as radiation therapy, may be dictated by the degree to which tubulogenesis has been activated. It is well known that the bulk of prostate cancer is relatively radiation resistant as compared to other tumor types. As a slow growing tumor, it is generally considered a tumor type that can be successfully treated using hypofractionation at fractional doses up to 2.8 Gy, since tumor repopulation is not a factor [80]. Other groups are testing whether hypofractionated stereotactic body radiation therapy (19.5 Gy in 3 fractions) followed by intensity-modulated radiation therapy (IMRT) (dose of 50.4 Gy in 28 fractions) offers radiobiological benefits of a large fraction boost for dose escalation. The goal is to achieve a well-tolerated treatment option for men with intermediate- to high-risk prostate cancer [81]. Understanding the biological responsiveness of invasive budding tumor cells and the extent of their activation as compared to the bulk of the tumor are likely to increase the biological effectiveness of the therapy and limit normal tissue damage. 

Preclinical xenograft and tissue culture studies revealed the phenomenon of cell adhesion-mediated radiation resistance (CAM-RR) [82–90]. CAM-RR can be overcome by the loss of tumor cell adhesion to the extracellular matrix [25, 86, 90, 91]. Since the tubulogenesis model of invasive cancers involves the loss of cell adhesion, one would predict that an increased efficacy of radiation therapy to block invasive tubulogenesis may be possible using lower doses and lower fractions of radiation therapy than is currently prescribed. As stated above, such an approach may prove more effective and potentially reduce damage to surrounding tissue.

## 3. Summary

The tubulogenesis model proposes that primary carcinomas of the prostate invade by a budding process similar to embryonic tubulogenesis. The majority of tumors arise from HGPIN lesions with the invasion occurring in portions of the gland where basal cells are lost, and the basal lamina is altered. If the tubulogenesis is complete, well-polarized tubules are formed which are recognized as low Gleason grade carcinoma; partial failure of polarity and lumen formation results in cribiform lesions; complete failure leads to the solid trabecular formations of Gleason grade 4 lesions. 

EMT is not observed in prostate carcinoma specimens either by direct morphological assessment or by immunohistochemical analysis of tissue using specific markers of EMT. If the EMT process does occur in the disease, it may occur as a late phenomenon most likely at the growing tips of the tubular structures. Lastly, a careful molecular analysis of the changes occurring at the tips of these tubular studies is likely to produce important information. Understanding molecular networks at the invasive tips may become the cornerstone of new diagnostic biomarkers to distinguish aggressive from indolent disease and to customize therapeutic treatments for preventing prostate carcinoma spread.
